# Association of neighbourhood residence and preferences with the built environment, work-related travel behaviours, and health implications for employed adults: Findings from the URBAN study

**DOI:** 10.1016/j.socscimed.2012.05.029

**Published:** 2012-10

**Authors:** Hannah M. Badland, Melody Oliver, Robin A. Kearns, Suzanne Mavoa, Karen Witten, Mitch J. Duncan, G. David Batty

**Affiliations:** a University of Melbourne, Australia; b Auckland University of Technology, New Zealand; c University of Auckland, New Zealand; d Massey University, New Zealand; e Central Queensland University, Australia; f University College London, UK

**Keywords:** Transport, Neighbourhood, Physical activity, Adults, New Zealand, Employed, Travel behaviour

## Abstract

Although the neighbourhoods and health field is well established, the relationships between neighbourhood selection, neighbourhood preference, work-related travel behaviours, and transport infrastructure have not been fully explored. It is likely that understanding these complex relationships more fully will inform urban policy development, and planning for neighbourhoods that support health behaviours. Accordingly, the objective of this study was to identify associations between these variables in a sample of employed adults. Self-reported demographic, work-related transport behaviours, and neighbourhood preference data were collected from 1616 employed adults recruited from 48 neighbourhoods located across four New Zealand cities. Data were collected between April 2008 and September 2010. Neighbourhood built environment measures were generated using geographical information systems. Findings demonstrated that more people preferred to live in urban (more walkable), rather than suburban (less walkable) settings. Those living in more suburban neighbourhoods had significantly longer work commute distances and lower density of public transport stops available within the neighbourhood when compared with those who lived in more urban neighbourhoods. Those preferring a suburban style neighbourhood commuted approximately 1.5 km further to work when compared with participants preferring urban settings. Respondents who preferred a suburban style neighbourhood were less likely to take public or active transport to/from work when compared with those who preferred an urban style setting, regardless of the neighbourhood type in which they resided. Although it is unlikely that constructing more walkable environments will result in work-related travel behaviour change for all, providing additional highly walkable environments will help satisfy the demand for these settings, reinforce positive health behaviours, and support those amenable to change to engage in higher levels of work-related public and active transport.

## Highlights

► More people preferred to live in urban (more walkable), rather than suburban (less walkable) neighbourhoods. ► Those living in less walkable neighbourhoods had longer commute distances to work. ► Living in more walkable neighbourhoods was associated with higher public transport access and lower automobile availability. ► Preferring suburban neighbourhoods was associated with lower levels of work-related public transport and active travel.

## Introduction

Where people choose or are required to live is a dynamic and multi-faceted construct with many factors underpinning the decision. ‘Place’ factors such as the availability and affordability of housing, school siting, employment locations, and public transport availability may be important considerations, while at the individual- and household-level, ‘people’ factors, such as raising a family, employment status, household income, mobility requirements, and responsibilities for care may influence area preference and choices/necessities (Cummins et al., 2007). Not withstanding the fact that people are frequently limited by their financial resources, conceptually these factors are aligned with the three tenets of the residential self-selection hypothesis (Chatman, 2009); first, households choose their location based on their travel preferences and anticipated commute patterns; second, neighbourhood characteristics and preferences are highly correlated; and third, those who strongly prefer a certain type of neighbourhood are more responsive to the built environment attributes of that setting. Yet, preference and selection issues are largely overlooked in the neighbourhood and health literature, potentially biasing area-level findings (Boone-Heinonen et al., 2011). Furthermore, it remains unknown how work-related travel behaviours are influenced by neighbourhood residence and preference.

Putting neighbourhood preference aside, established evidence suggests it is beneficial for health and community wellbeing to live in more walkable neighbourhoods (Bean et al., 2008; du Toit et al., 2007; Kawachi & Subramanian, 2007; Sallis et al., 2006). Such environments have street networks that are better connected, higher residential and employment densities, more diverse land uses and easier access to public transport (Sallis et al., 2006). There is a suggestion that people residing in these environments tend to walk more for transport purposes and have reduced reliance on automobiles for travel (du Toit et al., 2007), and, perhaps as a consequence they also report higher levels of physical activity, and greater neighbourhood cohesion and social interactions when compared with those who live in less walkable neighbourhoods (du Toit et al., 2007; Kawachi & Subramanian, 2007). For example, in suburban settings, distances between residents’ homes and many daily destinations, such as place of employment, are often greater than those observed in urban environments (du Toit et al., 2007). Inherently this leads to greater reliance on automobiles to meet daily transport needs within these settings. On one hand, cars afford increased mobility and the ability to travel independently to diverse and remote destinations, as well as encouraging flexibility in social networks and allowing compression of time. On the other hand, commuting by car may isolate individuals from the environment they travel through by removing the human negotiation and interaction that is required when travelling by other modes, leading to a reduction in unstructured and passing neighbourhood-level social interactions (Bean et al., 2008).

Physical activity patterns have been compared across diverse neighbourhoods. Rodriquez et al. (2006) found that the total volume of self-reported physical activity for residents living either in urban (more walkable) or conventional suburban neighbourhoods (less walkable) in the United States (**US**) did not actually differ. However, adults living in the more walkable neighbourhoods spent the most time engaged in physical activities within their neighbourhood and undertook higher levels of transport-related physical activity when compared with residents from less walkable neighbourhoods (Rodriquez et al., 2006). In addition, Handy et al. (2006) examined whether neighbourhood self-selection and travel attitudes mediated the relationship between walking and the built environment. Relationships between neighbourhood designs and self-reported walking behaviours of residents who had moved into the area within the previous year were examined. In the cross-sectional models, those who lived in more walkable neighbourhoods walked more frequently and for longer, and importantly, held more supportive attitudes towards walking, biking, and public transport than residents who lived in suburban settings. When the data were examined retrospectively, those who had a more positive attitude to walking had either a smaller decrease or a larger increase in walking when they moved between different neighbourhood types. In addition, substantial changes (i.e., 4 standard deviation points) in built environment factors were needed to alter walking behaviour. Based on these findings, the authors identified a built environment effect on walking that was independent of neighbourhood preference and travel attitudes (Handy et al., 2006), although preferences (as suggested by the residential self-selection hypothesis) remained important correlates for physical activity behaviours and attitudes.

Building on these findings, neighbourhood self-selection and preferences have been examined simultaneously in conjunction with walking for all purposes, vehicle miles travelled, and body size in US adults (Frank et al., 2007). Those who lived in, and preferred a more automobile dominant environment (i.e., less walkable), made more car trips, and the inverse was observed for those who preferred and lived in more urban (i.e., more walkable) neighbourhoods. Overall, 25% of the sample was mismatched; that is residents were not living in the neighbourhood context they preferred. Although not investigated in the study, possible explanations for the ‘mismatched’ findings are that neighbourhood selection at the individual level may be constrained by housing availability or adequate income sources to purchase in preferred neighbourhoods. Interestingly, however, neighbourhood preferences seemed to override a portion of the location effect. After controlling for demographic factors, those who preferred a more urban neighbourhood but lived in a suburban neighbourhood demonstrated higher levels of walking when compared with others who lived in and preferred suburban neighbourhoods. Those who preferred suburban neighbourhoods walked the least regardless of what type of neighbourhood they lived in.

Taken together, these findings highlight the importance of considering both area preference and residential selection when attempting to understand of how neighbourhood exposures relate to specific health behaviours and outcomes. As such, in this study we seek to examine associations between neighbourhood residence, preferences, and work-related travel behaviours and infrastructure in a sample of employed adults. To date the relationships between neighbourhood selection, neighbourhood preference, and work travel behaviours have not been explicitly examined, yet commuting to work contributes a substantial portion of all journeys made by adults (US Department Transportation and Bureau of Transportation Statistics, 2003), and walking and cycling to work have been associated with positive health gains (Andersen et al., 2000; Badland & Schofield, 2008a, 2008b). Commute distance to work (Badland et al., 2007), public transport access (Chen et al., 2008), and private motor vehicle (**PMV**) access (Badland & Schofield, 2008a, 2008b) have shown to be important predictors of work travel modes. It is unknown how these are related to neighbourhood selection and preference. As such, the aim of this study is to examine the relationships between neighbourhood residence, preference, transport infrastructure, and work-related travel behaviours in an employed sample of New Zealand adults. It is likely that understanding these complex relationships more fully will inform urban policy development through planning or retro-fitting of neighbourhoods to support healthy behaviours, namely transport-related physical activity.

## Methods

### Participants and setting

This study uses a sub-sample of data drawn from the Understanding the Relationship between Activity and Neighbourhoods (**URBAN**) study conducted in New Zealand. New Zealand has a population of 4.2 million residents, with 85% of the population living in urban/suburban settings, and has many physical and cultural similarities to Australia and North America (Statistics New Zealand, 2009). Detailed recruitment methodology is described elsewhere (Badland et al., 2009).

Briefly, data were collected between April 2008 and September 2010 when trained interviewers identified participants aged 20–65 years from selected neighbourhoods using a pre-determined door-to-door recruitment strategy. Forty-two households were randomly selected per neighbourhood, with one usually resident adult surveyed per household. This recruitment process was repeated in 12 selected neighbourhoods across four cities (totalling 48 neighbourhoods) in New Zealand, being Waitakere and North Shore (Auckland), Wellington, and Christchurch. For this study, only adults engaged in full- or part-time employment were included, resulting in the mean number of respondents per neighbourhood being 33.7 (range 24–43 residents).

Within each city, neighbourhoods comprising five contiguous mesh-blocks (smallest census area unit) were identified and selected on two constructs: ‘walkability’ (high/low) and population density of Māori residents (the indigenous population) (high Māori/low Maori). At a population level Maori experience both lower socio-economic status and poorer health outcomes (Ajwani et al., 2003). The rationale for selecting according to high/low Maori population density was to increase the proportion of Maori in the sample and the explanatory power of Maori specific-analyses. The walkability measure was generated using geographical information systems (**GIS**) to create neighbourhood-level indices based on street connectivity, dwelling density, land use mix, and retail floor area ratio. This measure is described in more detail elsewhere (Badland et al., 2009) and has been used in other countries to examine relationships between neighbourhood design and physical activity (Owen et al., 2007). The distribution of usual Māori residents domiciled in the neighbourhoods was estimated using 2006 census data. For each city, neighbourhood selection was as follows: 3× high walkable, high Māori neighbourhoods; 3× high walkable, low Māori neighbourhoods; 3× low walkable, high Māori neighbourhoods; and 3× low walkable, low Māori neighbourhoods. Neighbourhood selection was dichotomised as high or low walkability, and termed ‘neighbourhood residence’. All participants provided informed consent and ethical approval to conduct the study was provided by the host institution research ethics committee.

### Measures

Once recruited into the study, participants completed a computer-assisted personal interview (**CAPI**) with a trained interviewer. The face-to-face survey interview lasted approximately 40 min and covered various topics; the items relevant to this study included individual- and household-level demographics, employment status, primary workplace physical address, number of registered automobiles available within the household (a measure of private motor vehicle access), and neighbourhood preference. Many items and scales were taken from existing surveys that have been tested for reliability and validity as indicated elsewhere (Badland et al., 2009); however, aside from pilot testing, the URBAN study survey in its entirety has not undergone any formal reliability or validity testing.

#### Neighbourhood preference

Neighbourhood preference was assessed using items developed by Levine et al. (2005). Assuming housing cost, quality of schools, and mix of people were similar in both neighbourhoods, participants were asked to identify whether they would prefer to live in a more suburban or urban environment. A show card with ‘urban’ and ‘suburban’ images drawn was presented to participants alongside the verbal descriptions of hypothetical neighbourhoods.

The suburban neighbourhood was verbally described as being convenient for driving; with most destinations being a 10–15 min drive away from home, and place of work being within a 20-min commute on a motorway (freeway). This hypothesised neighbourhood did not support walking or public transport journeys to work. Dwellings were solely single-family houses on larger sections. In contrast, the hypothesised urban neighbourhood was verbally described as having good public transport and walking infrastructure with destinations (shopping, entertainment, libraries) being a 10–15 min walk away. Commute destinations were 20 min by public transport. Dwellings were close together and were a mixture of apartments, town houses, and small single-family houses on smaller parcel lots.

After selecting their preferred neighbourhood (suburban style or urban style), participants rated strength of preference on a five-point Likert scale (ranging from 1 = very slight preference to 5 = very strong preference, later collapsed to ‘no strong preference’ (1, 2) and ‘strong preference’ (3, 4, 5)). Derived variables were formed, being: urban style, no strong preference; suburban style, no strong preference; urban style, strong preference; and suburban style, strong preference. These derived variables were combined with the high/low walkability neighbourhood residence classifications.

#### Work-related travel modes, commute distance, and public transport access

Participants completed a trip diary for work-related journeys in the seven days prior to survey administration. Respondents self-reported their primary travel mode taken to and from their main place of work for each day. This was defined as the mode used for the greatest distance during the journey. To account for the variation in the days worked, the number of trips made by each travel mode (car, active travel (walking, cycling), and public transport) was calculated as a percentage of total work trips made during this period. Home to work commute distance was determined by geocoding participant’s home and primary workplace addresses using GIS. The closest facility function in ArcView v 9.2. software (ESRI, Redlands, CA) was used to model each participant’s shortest street network commute between his or her home and primary work address (Badland et al., 2008). The number of public transport (bus and train) stops inside each neighbourhood was identified on a GIS map overlay using ArcView v. 9.2. Neighbourhood boundaries were buffered by 20 m to capture any peripheral public transport stops. A summed score of public transport stops was derived for each neighbourhood and divided by the size of the neighbourhood area to create a measure of public transport density.

### Statistical analysis

Firstly, Spearman correlations were conducted to identify associations between demographic differences and neighbourhood residence, neighbourhood preferences, and a combined measure of these two variables. Secondly, linear regression models compared neighbourhood residence and neighbourhood preference with workplace commute distance, neighbourhood public transport density, and PMV access. Thirdly, logistic regression analyses were employed to examine the association between work travel modes compared by neighbourhood residence and neighbourhood preference. All regression models were adjusted for potential confounders, being sex, age, ethnicity, education, household income, housing tenure, and residential neighbourhood clustering (using robust standard errors). All analyses were conducted using Stata SE v.12 IC (StataCorp, College Station, TX) and *p*-values of less than 0.05 were considered statistically significant. This study was powered to detect the smallest between neighbourhood change in *r*^2^ of ≤2.3% and logistic regression models odds ratio (OR) of ≤1.27.

## Results

Overall, 1616 adults participated in this study. Findings shown in Table 1 identified differences in demographic characteristics when neighbourhood residence was compared; significant differences existed within age and housing tenure groups, with those preferring an urban setting being younger and living in rented dwellings. Across the sample, more people preferred to live in an urban, rather than suburban environment.

When neighbourhood residence and preference were combined (Table 2), significant differences existed within age, education attainment, and housing tenure groups. More owner–occupiers lived in low walkable neighbourhoods. Those who preferred an urban environment, regardless of where they lived, had the highest levels of education. After excluding those with no strong preference, approximately 43% of the sample was mismatched, with 26% of respondents preferring to live in a more urban style environment.

Differences in neighbourhood walkability and preferences by household- and neighbourhood-level features that were likely to influence travel behaviours are shown in Table 3. Employed adults who lived in less walkable neighbourhoods had significantly longer commute distances to their place of work than those living in more walkable neighbourhoods. Those who preferred a suburban style neighbourhood had an approximately 1.5 km further commute distance to their place of work when compared with participants who preferred urban settings. Respondents living in, and preferring suburban settings commuted 2.7 km extra to their place of work when compared with those who lived in and preferred an urban environment. Public transport stop density was greater in more walkable neighbourhoods and for those who preferred more suburban environments or had no strong preference had significantly less access to public transport than those preferring more urban neighbourhoods. As expected, those who lived in, or preferred suburban environments had greater automobile access.

Neighbourhood residence, neighbourhood preference, and a combined measure of these were significantly associated with the proportion of work trips made by car, public transport, and active travel (Table 4). Neighbourhood residence was significantly related to public transport and active transport work-related trips and these associations were in the expected direction. In the logistic regression models, the only significant relationship for car travel existed for those who lived in a low walkable neighbourhood, and had no neighbourhood preference. This group was more likely to commute by car compared with the group living in high walkable neighbourhoods with an urban style preference. Those who preferred a suburban style neighbourhood were less likely to take public or active transport to/from work when compared with those who preferred an urban setting. Similarly, those who lived in low walkable neighbourhoods, and preferred suburban style settings were less likely to travel using public transport or active transport, and these relationships were stronger than when the preference data were considered alone.

## Discussion

The aims of this study were to: gain a deeper understanding of the complex relationships between neighbourhood residence, preferred neighbourhood types, demographic variables, and environmental factors, and to understand how these were associated with work-related travel behaviours. Consistent with the residential self-selection hypothesis, our findings showed that 57% of the sample reported a ‘matched’ strong preference for the type of neighbourhood they lived in; however, 26% of the sample preferred to live in a more walkable environment than where they were current residing (compared with 17% preferring more suburban settings). Similar findings have been reported elsewhere (Frank et al., 2007). This mismatch may be due to a lack of more walkable neighbourhoods being available within New Zealand cities, particularly considering that the mismatch was found across all demographic and socio-economic groups. Furthermore, the respondent profile matched the neighbourhood demographic profile, so there is confidence that our findings are representative. It is important to reiterate, however, that respondents were asked to ignore important issues such as housing cost, schooling, and mix of people in selecting their preferred neighbourhood. This may have confounded the findings.

As expected, distance to work was shorter and the number of cars available in the household fewer for those who were living in more walkable neighbourhoods. Also, public transport stop density was greater in neighbourhoods with higher walkability, and those who rented were more likely to live in high walkable neighbourhoods. We do acknowledge, however, that the absolute number of public transport stops in the neighbourhood may not be the optimal measure of public transport access; service frequency, route variation, and access to meaningful destinations may be more relevant (Stone & Mees, 2010), yet we were unable to source this spatial data layer. It could be that people choose, in part, to live in suburban environments because these environments facilitate and reinforce car travel over other existing travel modes; for example, suburban neighbourhoods will likely have greater opportunity for unrestricted on-road and garage car-parking availability when compared with urban settings. These relationships were also reflected in the ‘no strong preference’ groups. The lack of associations between the reference group (high walkable neighbourhoods with an urban style preference) and those who lived in a low walkable neighbourhood, but preferred an urban style environment suggests that the attitudinal effects may at least in part mediate work-related travel behaviours.

A culture of car ownership exists in New Zealand, with 89.9% of households having access to at least one car (Statistics New Zealand, 2001); this compares to 92.1% of households in the US (US Department Transportation and Bureau of Transportation Statistics, 2003) and 73.0% of households in the United Kingdom (Office for National Statistics, 2011). This hegemony in car access likely hindered the ability to find more definitive relationships between neighbourhood residence, neighbourhood preference variables, and work-related automobile travel in our sample. Future research may benefit from assessing preferred mode of travel and travel demand modelling. In particular, work that identifies what features or interventions would encourage people residing in low walkable (but high public transport access) neighbourhoods to shift to public or active transport modes from car travel would be worthwhile, particularly considering that commute distances were not excessive for this sample irrespective of neighbourhood type. An avenue for this work may be to focus on identifying and reducing the time and monetary cost, as well as improving the service of public transport modes (either perceived or actual) when compared with car use (Wang, 2011).

### Limitations

When considering the contribution to knowledge, this study has identified relationships between neighbourhood residence, neighbourhood preferences, and work-related travel behaviours in a large representative sample of employed adults. As this is an observational (cross-sectional) study, causality and directionality cannot be inferred, and the investigation is limited by self-reported travel behaviours. Although we controlled for neighbourhood clustering and socio-economic position, our analysis approach may have over- or underestimated some of the associations identified. We did not attempt to tease out how specific sociodemographic indicators were related to PMV access, public transport use, or residential location choice and complexity in the first instance. These variables are influenced by socio-economic status (Dargay, 2001; Mutchler & Krivo, 1989), and as such, may have impacted on the relationships evident. Neighbourhoods were defined on the basis of New Zealand Statistics-derived administrative units. This synthetic application of aggregating administrative spatial units into neighbourhoods may or may not reflect how respondents define their neighbourhood, potentially over- or under-estimating the effect work travel behaviours have with neighbourhood preference. Furthermore, employment status and job type are important contributors in determining residential location, neighbourhood preference, and mode of travel to work, and these were not examined in any detail in this study. Further consideration of these factors alongside understanding neighbourhood selection, neighbourhood social constructs, travel mode preferences, and travel demand modelling is now needed. Neighbourhood preferences now need to be examined beyond the hypothetical construct utilised here.

## Figures and Tables

**Table 1:** Demographic profile of respondents stratified by neighbourhood residence and preference.

Characteristic	Neighbourhood residence^a^			Neighbourhood preference^a^			
	High walkability *n* = 811 %	Low walkability *n* = 805 %	*p*-Value	Urban style preference *n* = 817 %	Suburban style preference *n* = 562 %	No strong preference *n* = 217 %	*p*-Value
Sex			*0.09*				*0.46*
Male	42.8	47.1		46.1	42.9	45.2	

Age (years)			*≤0.01*				*0.60*
16–24	10.7	7.9		9.8	7.4	12.0	
25–34	22.9	19.2		22.3	18.9	22.6	
35–44	26.6	28.1		25.7	30.6	25.3	
45–55	24.0	26.8		25.7	26.1	22.1	
55–65	15.8	18.1		16.5	17.1	18.0	

Ethnicity			*0.86*				*0.53*
Maori/Polynesian	14.7	15.5		14.0	17.6	13.8	
Asian	10.4	9.7		9.7	11.4	8.8	
NZ European & other	75.0	74.8		76.4	71.0	77.4	

Education attainment			*0.36*				*≤0.001*
Finished primary school	3.0	4.5		2.2	5.7	4.6	
Finished high school	20.8	20.7		18.5	24.4	21.7	
University Entrance	10.0	10.7		9.5	10.7	11.1	
Apprenticeship/diploma	24.8	23.9		22.3	27.4	25.3	
Degree or higher	40.9	40.2		47.7	31.3	37.3	

Annual household income (NZD)			*0.99*				*≤0.05*
≤$20,000	3.8	2.9		4.4	1.6	4.1	
$20,001–$40,000	11.1	11.2		11.3	11.0	12.0	
$40,001–$60,000	16.3	15.7		16.2	15.8	14.3	
$60,001–$80,000	13.9	15.4		14.9	15.3	12.4	
$80,001–$100,000	14.9	16.0		14.9	17.3	13.4	
≥$100,001	30.8	30.4		31.7	28.5	32.3	

Housing tenure			*≤0.001*				*≤0.001*
Owner–occupier	54.8	66.5		54.5	66.7	66.8	

*Key*: NZD = New Zealand dollars; SD = standard deviation.

a Percentages do not equal 100% because of missing data.

**Table 2:** Demographic profile of respondents stratified by a combined measure of neighbourhood residence and neighbourhood preference.

Characteristic	Neighbourhood residence × neighbourhood preference^a^						
	High walkability urban style preference *n* = 465 %	High walkability suburban style preference *n* = 240 %	High walkability no strong preference *n* = 100 %	Low walkability urban style preference *n* = 352 %	Low walkability suburban style preference *n* = 322 %	Low walkability no strong preference *n* = 117 %	*p*-Value
Sex							*0.28*
Male	43.9	40.8	42.0	49.1	44.4	47.9	

Age (years)							*0.01*
16–24	10.6	8.9	15.0	8.8	6.3	9.4	
25–34	22.3	24.1	23.0	22.2	15.0	22.2	
35–44	26.2	29.1	24.0	25.0	31.7	26.5	
45–55	23.0	25.3	23.0	29.3	26.6	21.4	
55–65	17.8	12.7	15.0	14.8	20.4	20.5	

Ethnicity							*0.07*
Maori/Polynesian	13.5	17.9	12.0	14.5	17.4	15.4	
Asian	9.2	12.9	10.1	10.2	10.2	7.7	
NZ European + other	77.2	69.2	78.0	75.3	72.4	76.9	

Education attainment							*≤0.01*
Finished primary school	2.4	4.6	2.0	2.0	6.5	6.8	
Finished high school	17.0	27.5	23.0	20.5	22.0	20.5	
University entrance	10.1	10.4	8.0	8.8	10.9	13.7	
Apprenticeship/diploma	22.4	26.3	32.0	22.2	28.3	19.7	
Degree or higher	48.0	30.0	35.0	46.6	32.3	39.3	

Annual household income (NZD)							*0.35*
≤$20,000	4.5	1.7	6.0	4.3	1.6	2.6	
$20,001–$40,000	10.8	12.5	10.0	11.9	9.9	13.7	
$40,001–$60,000	15.3	17.5	16.0	17.3	14.6	12.8	
$60,001–$80,000	14.8	12.1	15.0	15.1	17.7	10.3	
$80,001–$100,000	13.5	19.2	11.0	16.8	15.8	15.4	
≥$100,001	33.3	26.3	30.0	29.5	30.1	34.2	

Housing tenure							*≤0.001*
Owner–occupier	52.5	57.3	59.0	57.1	73.6	73.5	

*Key*: NZD = New Zealand dollars; SD = standard deviation.

a Percentages do not equal 100% because of missing data.

**Table 3:** Comparison of distance to workplace, public transport stop density, and private automobile ownership by neighbourhood residence and neighbourhood preference.

Condition	Shortest street network distance to primary workplace (km)					
	Mean ± SD	*p*-Value	Coef^a^	95% CI	Robust SE	*p*-Value
Neighbourhood residence						
High walkability	6.4 ± 7.3	≤0.01	ref			
Low walkability	7.4 ± 7.3		0.95	−0.58 to 2.48	0.76	0.22

Neighbourhood preference		≤0.001				
Urban style preference	6.2 ± 6.8		ref			
Suburban style preference	7.7 ± 8.0		1.57	0.422–2.71	0.57	≤0.01
No strong preference	6.9 ± 6.9		0.72	−0.48 to 1.92	0.60	0.23

Neighbourhood residence × neighbourhood preference		≤0.001				
High walkability urban style preference	6.2 ± 7.5		ref			
High walkability suburban style preference	6.6 ± 7.3		0.39	−0.93 to 1.71	0.66	0.56
High walkability no strong preference	6.3 ± 6.5		0.00	−1.68 to 1.65	0.83	0.99
Low walkability urban style preference	6.1 ± 5.8		0.00	−2.14 to 1.90	1.00	0.91
Low walkability suburban style preference	8.6 ± 8.4		2.35	0.48–4.23	0.93	≤0.05
Low walkability no strong preference	7.5 ± 7.2		1.25	−0.60 to 8.47	0.92	0.18


Condition	Density of public transport stops per km^2^ of neighbourhood					
	Mean ± SD	*p*-Value	Coef^a^	95% CI	Robust SE	*p*-Value
Neighbourhood residence		≤0.001				
High walkability	0.05 ± 0.04		ref			
Low walkability	0.04 ± 0.03		−0.01	−0.03 to 0.01	0.01	0.15

Neighbourhood preference		≤0.001				
Urban style preference	0.05 ± 0.04		ref			
Suburban style preference	0.04 ± 0.03		−0.01	−0.02 to 0.00	0.01	≤0.01
No strong preference	0.04 ± 0.03		−0.01	−0.02 to 0.00	0.00	≤0.01

Neighbourhood residence × neighbourhood preference		≤0.001				
High walkability urban style preference	0.06 ± 0.04		ref			
High walkability suburban style preference	0.05 ± 0.04		−0.01	−0.03 to 0.01	0.01	0.24
High walkability no strong preference	0.04 ± 0.02		−0.01	−0.03 to 0.00	0.01	≤0.05
Low walkability urban style preference	0.03 ± 0.02		−0.01	−0.03 to 0.01	0.01	0.30
Low walkability suburban style preference	0.03 ± 0.02		−0.03	−0.05 to 0.00	0.01	≤0.05
Low walkability no strong preference	0.04 ± 0.02		−0.02	−0.04 to 0.00	0.01	0.07


Condition	Number of private automobiles in household					
	Mean ± SD	*p*-Value	Coef^a^	95% CI	Robust SE	*p*-Value
Neighbourhood residence		0.19				
High walkability	1.80 ± 1.06		ref			
Low walkability	3.2 ± 31.3		1.01	−0.37 to 2.39	0.68	0.15

Neighbourhood preference		≤0.001				
Urban style preference	1.75 ± 1.12		ref			
Suburban style preference	2.24 ± 1.23		−0.09	−1.38 to 1.19	0.64	0.88
No strong preference	1.98 ± 1.19		0.28	−0.21 to 0.76	0.24	0.26

Neighbourhood residence × neighbourhood preference		≤0.001				
High walkability urban style preference	1.68 ± 1.07		ref			
High walkability suburban style preference	2.05 ± 1.08		0.76	−0.23 to 1.74	0.49	0.13
High walkability no strong preference	1.72 ± 0.88		0.27	−0.56 to 1.09	0.41	0.52
Low walkability urban style preference	1.85 ± 1.17		0.80	−0.45 to 2.04	0.62	0.21
Low walkability suburban style preference	2.38 ± 1.32		−0.13	−2.13 to 1.87	0.99	0.90
Low walkability no strong preference	2.21 ± 1.37		0.92	−0.09 to 1.93	0.50	0.07

Key: CI = confidence interval; Coef = coefficient; km = kilometres; m = metres; ref = reference category; SD = standard deviation; SE = standard error.

a Regression models adjusted for sex, age, ethnicity, education attainment, household income, housing tenure, and neighbourhood clustering.

**Table 4:** Comparison of work-related travel modes by neighbourhood residence and neighbourhood preference.

Condition	Work-related car travel commute					
	% of All work trips	*p*-Value	OR^a^	95% CI	Robust SE	*p*-Value
Neighbourhood residence		≤0.01				
High walkability	64.1		ref			
Low walkability	70.5		1.17	0.85–1.60	0.19	0.35

Neighbourhood preference		≤0.001				
Urban style preference	61.5		ref			
Suburban style preference	73.8		1.23	0.92–1.65	0.18	0.16
No strong preference	73.0		1.47	0.95–2.28	0.33	0.08

Neighbourhood residence × neighbourhood preference		≤0.001				
High walkability urban style preference	60.4		ref			
High walkability suburban style preference	72.4		1.30	0.85–1.98	0.28	0.23
High walkability no strong preference	61.6		0.93	0.56–1.55	0.24	0.79
Low walkability urban style preference	63.1		1.04	0.71–1.54	0.21	0.84
Low walkability suburban style preference	74.8		1.22	0.79–1.90	0.28	0.37
Low walkability no strong preference	82.7		2.67	1.33–5.34	0.95	≤0.01


Condition	Work-related public transport commute					
	% of All work trips	*p*-Value	OR^a^	95% CI	Robust SE	*p*-Value
Neighbourhood residence		0.08				
High walkability	8.7		ref			
Low walkability	6.8		0.79	0.46–1.35	0.22	0.38

Neighbourhood preference		≤0.01				
Urban style preference	9.5		ref			
Suburban style preference	6.3		0.54	0.38–0.76	0.10	≤0.001
No strong preference	5.2		0.48	0.29–0.79	0.12	≤0.05

Neighbourhood residence × neighbourhood preference		≤0.05				
High walkability urban style preference	9.8		ref			
High walkability suburban style preference	6.8		0.55	0.33–0.89	0.14	≤0.05
High walkability no strong preference	7.9		0.64	0.33–1.24	0.22	0.19
Low walkability urban style preference	9.0		0.93	0.48–1.80	0.31	0.84
Low walkability suburban style preference	6.0		0.50	0.29–0.88	0.14	≤0.05
Low walkability no strong preference	2.3		0.33	0.15–0.75	0.14	≤0.01


Condition	Work-related active transport commute					
	% of All work trips	*p*-Value	OR^a^	95% CI	Robust SE	*p*-Value
Neighbourhood residence		≤0.001				
High walkability	16.0		ref			
Low walkability	11.0		0.66	0.30–1.46	0.27	0.31

Neighbourhood preference		≤0.001				
Urban style preference	18.4		ref			
Suburban style preference	7.6		0.36	0.23–0.55	0.08	≤0.001
No strong preference	9.9		0.58	0.34–0.98	0.16	≤0.05

Neighbourhood residence × neighbourhood preference		≤0.001				
High walkability urban style preference	20.3		ref			
High walkability suburban style preference	9.5		0.38	0.21–0.70	0.12	≤0.01
High walkability no strong preference	11.5		0.57	0.29–1.15	0.20	0.12
Low walkability urban style preference	16.0		0.74	0.28–1.97	0.37	0.55
Low walkability suburban style preference	6.2		0.26	0.11–0.62	0.11	≤0.01
Low walkability no strong preference	8.5		0.45	0.19–1.06	0.19	0.07

Key: CI = confidence interval; km = kilometres; m = metres; OR = odds ratio; ref = reference category; SD = standard deviation; SE = standard error.

a Regression models adjusted for sex, age, ethnicity, education attainment, household income, housing tenure, and neighbourhood clustering.
